# Socioeconomic and geographic variations of disabilities in India: evidence from the National Family Health Survey, 2019–21

**DOI:** 10.1186/s12942-024-00363-w

**Published:** 2024-02-18

**Authors:** Rashmi Rashmi, Sanjay K. Mohanty

**Affiliations:** Department of Population and Development, International Institute for Population Sciences, Mumbai, 400088 India

**Keywords:** Disability, Socioeconomic variation, Geographic variation, Cluster, District, Locomotor, Mental, Visual, Speech, Hearing, India

## Abstract

**Background:**

Increasing disability is of global and national concern. Lack of evidence on disability across socioeconomic groups and geographic levels (especially small areas) impeded interventions for these disadvantaged subgroups. We aimed to examine the socioeconomic and geographic variations in disabilities, namely hearing, speech, visual, mental, and locomotor, in Indian participants using cross-sectional data from the National Family Health Survey 2019–2021.

**Methods:**

Using data from 27,93,971 individuals, we estimated age-sex-adjusted disability rates at the national and sub-national levels. The extent of socioeconomic variations in disabilities was explored using the Erreygers Concentration Index and presented graphically through a concentration curve. We adopted a four-level random intercept logit model to compute the variance partitioning coefficient (VPC) to assess the significance of each geographical unit in total variability. We also calculated precision-weighted disability estimates of individuals across 707 districts and showed their correlation with within-district or between-cluster standard deviation.

**Results:**

We estimated the prevalence of any disability of 10 per 1000 population. The locomotor disability was common, followed by mental, speech, hearing, and visual. The concentration index of each type of disability was highest in the poorest wealth quintile households and illiterate 18 + individuals, confirming higher socioeconomic variations in disability rates. Clusters share the largest source of geographic variation for any disability (6.5%), hearing (5.8%), visual (24.3%), and locomotor (17.4%). However, States/Union Territories (UTs) account for the highest variation in speech (3.7%) and mental (6.5%) disabilities, where the variation at the cluster level becomes negligible. Districts with the highest disability rates were clustered in Madhya Pradesh, Maharashtra, Karnataka, Tamil Nadu, Telangana, and Punjab. Further, we found positive correlations between the district rates and cluster standard deviations (SDs) for disabilities.

**Conclusions:**

Though the growing disability condition in India is itself a concerning issue, wide variations across socioeconomic groups and geographic locations indicate the implementation of several policy-relevant implications focusing on these vulnerable chunks of the population. Further, the critical importance of small-area variations within districts suggests the design of strategies targeting these high-burden areas of disabilities.

**Supplementary Information:**

The online version contains supplementary material available at 10.1186/s12942-024-00363-w.

## Background

With the demographic and epidemiological transitions, disability has been on the rise [1, 2]. About 1.3 billion people have some form of disability worldwide [3]. Over 250 million people experience moderate or severe disabilities, and the difficulty performing daily activities is shared among 46% of the elderly  aged 60 years and above [4]. The prevalence of disability has increased from 10% in 1970 to 16% by 2023 [3]. In any age group, disability can cause massive suffering; however, such a significant public health issue has not received adequate attention yet [5].

The concept of disability has not been uniformly defined across countries. The World Health Organization (WHO) defines disability as the condition of the body or mind that makes it more difficult for a person to do certain activities and interact with the world around them [2]. Conceptual and definitional differences exist across censuses or surveys, even within countries. The consensus from varying definitions and estimates suggests that the disability rate has been rising across developing countries [2, 6]. India, too, had an increasing disability burden. The Census of India, India’s primary official source of nationwide disability statistics, showed an increment from 2.13% (21.91 million) in 2001 to 2.21% (26.81 million) in 2011 [7]. A World Bank report estimated that there are over 50–80 million disabled population in India [8, 9]. Such estimation variations occur primarily due to differences in definition, classification, and operationalization within the country [10].

With a population of 1,211 million, India is experiencing a rapid demographic and epidemiological transition [7]. The size of disabled people has increased, mainly due to the aged population [11]. Although locomotor disability remains high in all ages, it is more prevalent in working age groups (51%) [11]. Disability is often linked to an individual’s age, sex, and health condition [12–15]. However, little is known that disability and socioeconomic status are intricately linked phenomena in India [16, 17]. Persons with disabilities face lower educational attainment, lower employment, poor living condition, and decreased access to services [16, 18, 19]. Conversely, people with socioeconomic disadvantages may suffer worse health or intensified disability conditions [20–22]. Moreover, families living with disabled members have a higher chance of incurring catastrophic health expenditures, leaving them in poverty [23]. Ample evidence from India indicated socioeconomic variation of disability [14, 24–27]. Along with higher poverty and inequality in India, there was striking geographical variation in the level of development across states and districts [28, 29]. The lack of variability evidence on one of the neglected health outcomes, i.e., disability, further urges us to look into its variations across geographical dimensions. In this context, using the recent round of the National Family Health Survey (2019–2021), we aimed to examine the socio-economic and geographic variations of disabilities in India.

Following are the rationale of the present study: First, with limited research on the association of disability with socioeconomic gradient, socioeconomic disparities in disability, especially across its different types, are poorly understood in India [30]. The present study takes advantage of India’s latest nationally representative data, which provides information on disability types and relative poverty measures, i.e., wealth index [31], to contribute to India’s minimal disability research. Second, although efforts have been made to describe the extent of disability variation between states and regions of India [32, 33]. These literatures relied on the single geographic level and was confined to higher levels like state and National Sample Survey (NSS) regions, thus, left unrevealed the small area variations in disabilities occurring within districts and between clusters. It should be noted that the importance of any given unit could be better understood when all the possible geographical levels are thought to contribute to the large variation simultaneously [34]. Moreover, given the importance of small-area levels serving as a central policy unit in India, there is a need to explore the small-area variations in disabilities between and within geographical areas. Thus, the present study aims to elucidate socioeconomic and geographical variations of disabilities, especially focusing on the small area variations in disability by considering heterogeneity within districts and between clusters.

## Methods

### Study design and participants

We used unit data from the fifth round of the National Family Health Survey (NFHS) conducted between 2019 and 2021. NFHS is a large-scale multi-round survey providing comprehensive information on population, health, and nutrition across India and each state/union territory (UT). The recent round, i.e., NFHS-5, covered newer topics, such as preschool education, disability, access to a toilet facility, death registration, bathing practices during menstruation, and methods and reasons for abortion. The survey successfully interviewed 2,843,917 household members from 636,699 households, among which 724,115 women aged 15–49 years and 101,839 men aged 15–54 years, along with other age group people, using a multistage stratified sampling design. Detailed information on study design, sample, collection, and available findings are in the national report [35]. This study is based on secondary data available in the public domain; written informed consent was obtained from all participants during the survey. Therefore, there was no need to obtain ethical clearance for this study.

The final analytical sample included 27,93,971 individuals from 636,189 households nested within 30,170 clusters, 707 districts, and 36 states/union territories (Additional file 1: Figure S1). The final selection includes only the de jure population and excludes those having missing covariates. Additional file 1: Table S2 extensively provides the number of districts, clusters, total individuals, and disabled individuals nested within each of the 36 states/union territories. Uttar Pradesh, followed by Bihar, was the largest state with 75 and 38 districts, respectively. Among the individuals, 26,394 had some form of disability, further identified by their disability types.

### Variables

#### Outcome variable

The primary outcome variable was any form of disability. Those with any disability were categorized according to their disability type: hearing, speech, visual, mental, locomotor, and other.

NFHS-5 collected information on the disability status of individuals in the household questionnaire from the de-jure population using the following questions: “*Does any usual resident of your household, including you, have any disability?”.* The individual was identified with the line number in the household answering “yes,” which was further identified according to their disability types. The description of all kinds of disabilities is provided in Additional file 1: Table S3.

#### Independent variables

Socioeconomic status is shown through the relative poverty measure, i.e., wealth index [31]. The wealth index is constructed using principal component analysis (PCA) conducted on a set of 37 variables based on household amenities, assets, and consumer durables [36]. The wealth scores generated from the PCA are split into quintiles ranging from the poorest to the richest, with 20 percentiles each. The lowest 20 percent of households were coded as poorest and the highest 20 percent as richest. This was termed the wealth quintile in the analysis and was available in the dataset. The wealth index was computed separately for urban and rural areas.

Based on the prior literature, we included individual, demographic, and household variables as possible confounders [12, 14, 16, 21, 30]. Age is categorised as 0–9 years, 10–19 years, 20–29 years, 30–39 years, 40–49 years, 50–59 years, and 60 + years. Sex as male or female. Education of individuals categorised as no education, less than 5 years, 5–9 years, and 10 + years. Religions as Hindu, Muslim, Christian, and Others. Caste is classified as [Scheduled Caste/Scheduled Tribes] (SC/ST), [Other Backward Caste] (OBC) and Others. The residence is categorised into either rural or urban.

Taking cue from previous literature [37, 38], we included two standard cluster variables by aggregating the individual’s educational level and wealth status: (a) Education level of the cluster, defined as the proportion of individuals with more than 10 years of schooling among all individuals in the cluster. (b) Wealth status of cluster; is defined as the proportion of the rich and richest households among all the households in the cluster. We categorised the proportions of education level and wealth status of the cluster as low, medium, and high. It denotes that the higher the proportion of educated individuals, the higher the cluster’s educational standard. And the higher proportion of rich and richest households means higher wealth status in the cluster.

Similarly, we included two standard variables for districts and states, aggregating the educational level and wealth status. The variables are the Education level of district (low, medium, high), Wealth status of district (low, medium, high), Education level of state (low, medium, high), Wealth status of state (low, medium, high).

### Statistical analysis

Frequency and percentage distributions were used to show participant characteristics. Age -sex-adjusted disability rates (per 1000 population) were reported across the participant characteristics and states. All the analyses and figures presented were prepared in STATA software, and the *“syvset”* command was used in the study for accounting sampling weights, clustering, and stratification. To assess the socioeconomic inequality in the prevalence of disability (by its type), we measured the Erreygers Concentration Index (EI). It should be noted that the concentration indices are the popular choice of measuring socioeconomic-related health inequality, preferred over relative index and slope index of inequality [39]. The disproportionality-based relative measure of inequality (i.e., Relative Concentration Index, CI) is used to express inequality as a function of shares of the health indicator compared to shares of the population [40].

We used the CI as follows: ${\text{CI}}= \frac{2}{\mu } cov({y}_{i}, {r}_{i})$

where ${y}_{i}$ is an indicator of disability type for individual i, ${r}_{i}$ is the fractional ranking of individuals according to the wealth index, and $\mu$ is the mean of ${y}_{i}$ [41].

However, when the outcome variable is a binary indicator, the range of CI often shortens. So, for a more robust result, we have used Erreygers Concentration Index (EI) to address such a problem [42]. It is a normalized form of CI defined as

$${\text{EI}}\, = \,\frac{4\mu }{{b\, - \,a}}\,CI$$

Here, $\mu$ is the mean of the disability variable by its type, $CI$ is the standard CI, b is the maximum value of the disability variable (i.e., 1), and a is the minimum value of the disability variable (i.e., 0).

EI varies between −1 and + 1, with a negative value suggesting a concentration of disability among the poorest and a positive value indicating a concentration of disability among the richest.

Besides wealth, education is also a strong determinant of health, as educated people are more likely to use information effectively and efficiently, making them aware of their health and likely to follow medical advice [43]. So, we carried out sensitivity analyses in which the education of 18 + age individuals were also used to elucidate socioeconomic variations of disabilities. It should be noted that the educational variations were confined to 18 + adults as those in 0–17 years of age are mostly a dependent population who may or may not be responsible for their health decisions. Further, age-sex-adjusted estimates were calculated for disability rates in all inequality dimensions.

The present study used a visual way to illustrate the concentration index through the concentration curve. The concentration curve plots the cumulative percentage of disability variable (by type) on its y-axis against the cumulative percentage of household wealth index/individual education level on the x-axis. If the concentration curve lies above the line of perfect equality, the disability is concentrated among poor/uneducated people, and vice versa [44].

Multivariable analysis using a four-level random intercept logit model showed the risk factors associated with disability across its type, adjusting for all covariates (reporting coefficients with 95% confidence intervals). We adopted multilevel analysis to partition the total geographic variations for the probability of an individual *i* (level-1) in cluster *j* (level-2), district *k* (level-3) and state/UT *l* (level-4) having a disability (Y) using the equation:

$${\text{logit}}\left( {\Pr \left( {Y_{ijkl} \, = \,1{|}X} \right)} \right)\, = \,{\upbeta }_{0} \, + \,{\beta X}_{{{\text{ijkl}}}} \, + \,\left( {{\upmu }_{0jkl} \, + \,{\text{v}}_{0kl} \, + \,{ }f_{0l} } \right)$$

where the dependent variable Y (disability by its type) and set of independent covariates X were assumed to follow a four-level data structure. ${\upbeta }_{0}$ represents the constant and ${\upmu }_{0jkl},{{\text{v}}}_{0kl}{, f}_{0l}$ are residuals specific to cluster, district and state/UT, respectively. The residuals are assumed to be normally distributed with a mean of 0 and variances of ${{\sigma }^{2}}_{\upmu 0}$, ${{\sigma }^{2}}_{{\text{v}}0}$ and ${{\sigma }^{2}}_{{\text{f}}0}$. These variances estimate were between clusters within a district (${{\sigma }^{2}}_{\upmu 0}$), between districts within a state/UT (${{\sigma }^{2}}_{{\text{v}}0}$) and between states/UT within the country (${{\sigma }^{2}}_{{\text{f}}0}$), respectively. We assumed a fixed individual-level variance of $\frac{{\pi }^{2}}{3}$ or 3.29 due to binary nature of outcomes [45, 46]. The multilevel model was applied using MLwiN 3.05 software program via the “runmlwin” command in STATA 16.0 with default prior distributions of iterated generalized least square (IGLS) estimation method [47].

It should be noted that the two model specifications, namely the null model (without any covariates) and the adjusted model (controls all the covariates), were estimated based on the above modelling structure. We computed the variance partitioning coefficient (VPC) to assess the significance of each geographical unit in total variability for null and adjusted models, such as for unit z, VPC% = $\frac{{{\sigma }^{2}}_{{\text{Z}}}}{{{\sigma }^{2}}_{\upmu 0}+{{\sigma }^{2}}_{{\text{v}}0}+{{\sigma }^{2}}_{{\text{f}}0}+\frac{ {\pi }^{2}}{3}} * 100$. Further, we carried out a sensitivity analysis using two-level model structures in which individuals were assumed to be nested within only one geographic level. This helps to evaluate the changes in the variance estimates and the proportion of variance attributable to high levels from all four levels to only one geographic level at a time.

Next, we generated district-specific precision-weighted estimates for each type of disability. The probability of disability for each district was calculated as equation: $\frac{{\text{exp}}({\upbeta }_{0}+{{\text{v}}}_{0kl}+ {f}_{0l})}{1+{\text{exp}}({\upbeta }_{0}+{{\text{v}}}_{0kl}+ {f}_{0l})}$. The precision-weighted probability was further multiplied by 1000 for interpretation clarity. We also generated precision-weighted estimates specific to each cluster for disabilities, calculated as$\frac{{\text{exp}}({{\upbeta }_{0}+\upmu }_{0jkl} +{{\text{v}}}_{0kl}+ {f}_{0l})}{1+{\text{exp}}({{\upbeta }_{0}+\upmu }_{0jkl} +{{\text{v}}}_{0kl}+ {f}_{0l})}$. The within-district or between-cluster small area variations were computed as standard deviations (SDs) of these estimates. The district-level maps were prepared using QGIS 3.28 software. The shapefile was obtained from the DHS Spatial repository (https://spatialdata.dhsprogram.com/boundaries).

## Results

### Sample characteristics and outcomes

**Fig. 1:**
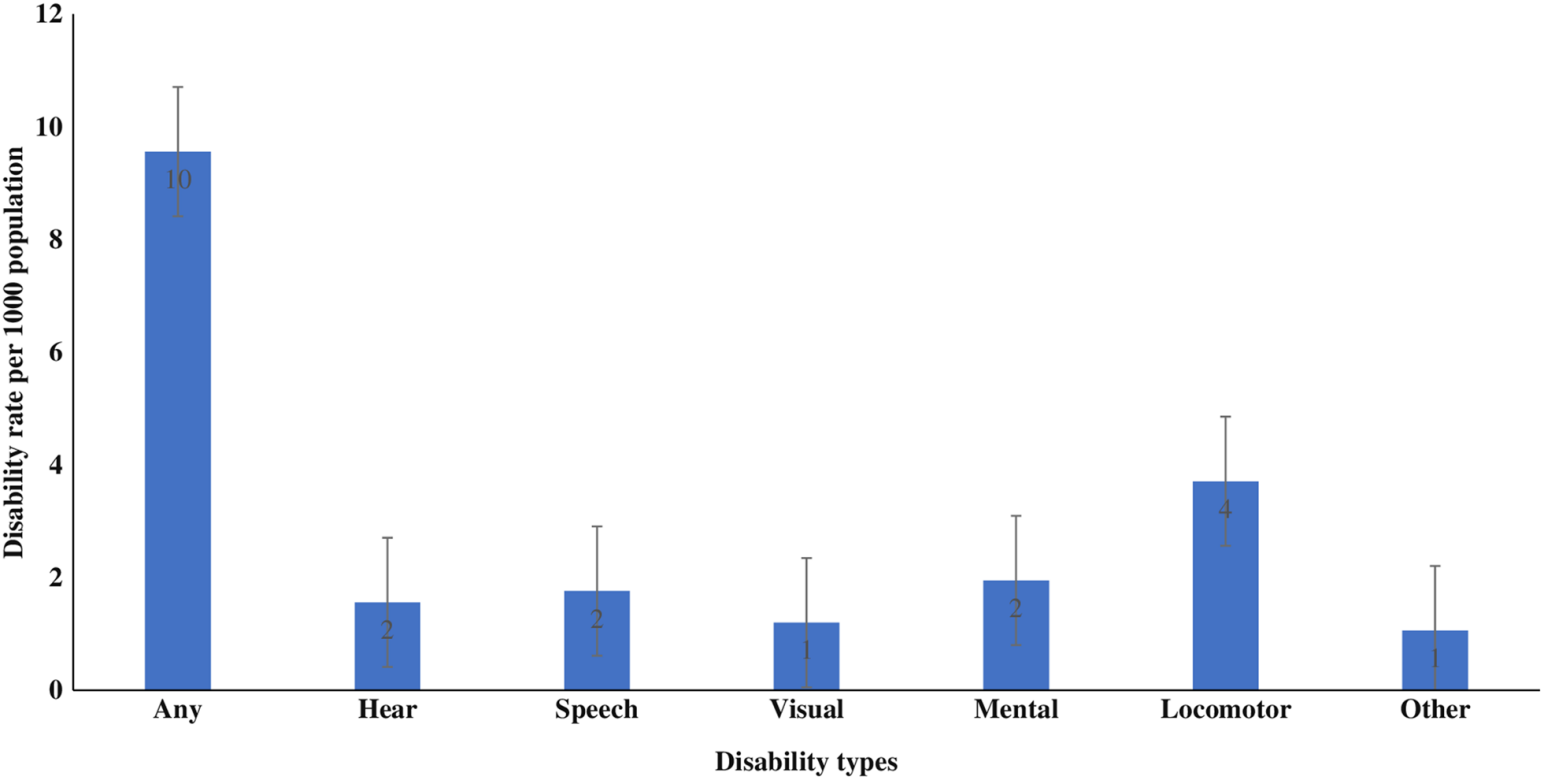
Disability rate per 1000 population, NFHS 2019–21, India

Additional file 1: Table S4 lists the characteristics of 27,93,971 participants in the study. About 88% of participants were under 60 years of age. Half the population was female (50.3%), and most participants resided in rural areas (68.2%). Different subgroups of the wealth quintile are divided equally among the population. Less than one-third of the participants have never received an education (30.7%). About 81% of the sample were Hindu, and 42% were in the OBC caste category. Districts with highest wealth status and educational level includes nearly 34% and 39% of individuals respectively. Further, 44.9% and 34.4% of individuals were from states with low educational level and lower wealth status, respectively. Figure 1 shows the disability rate (per 1000 population) by type in India. Approximately ten persons per thousand population have some form of disability. Across the disability types, the rate of locomotor disability was the highest (4 per 1000), followed by mental (2 per 1000), speech, hearing, and visual.

### Age-sex-adjusted disability (by type) across characteristics and states/UTs

Table 1 presents the age-sex-adjusted disabilities (per 1000 population) across participants’ characteristics in India. Overall, 9.5 per 1000 participants had a disability in any form. Among the types, locomotor disability was the highest. About 15 per 1000 participants aged 60 and above have at least one disability condition. The prevalence of different types of disability remains more elevated in the 60 + age group, except for speech and mental disability. Speech and mental disabilities were higher in the growing age groups (10–29 years). Male participants reported a higher prevalence of all types of disability. Those with no formal education and residing in rural areas show higher disabilities. All the types of disability, namely, hearing, speech, visual, mental, and locomotor, were common among participants living in the poorest wealth quintile households. Disabilities were higher in clusters and districts with low and medium educational level and wealth status. In contrast, states with individuals having higher educational levels and wealth status show more disabilities.

**Table 1:** Age-sex adjusted disabilities (per 1000 population) across participant characteristics in India, NFHS 2019–21

Participant characteristics	Type of disability						
	Any	Hearing	Speech	Visual	Mental	Locomotor	Other
Age of household members							
0–9	4.5	0.9	1.8	0.6	1.1	1.7	0.4
10–19	7.8	1.3	2.4	0.9	2.5	2.4	0.8
20–29	9.2	1.2	2.0	1.0	2.7	3.2	0.9
30–39	11.0	1.3	1.8	1.1	2.3	4.7	1.2
40–49	11.4	1.7	1.6	1.4	2.2	4.8	1.3
50–59	11.1	1.8	1.1	1.6	1.4	4.5	1.5
60 +	15.0	3.4	1.2	2.4	1.1	6.3	1.9
Sex of household members							
Male	11.6	1.7	2.0	1.4	2.3	4.8	1.4
Female	7.5	1.4	1.5	1.0	1.6	2.6	0.8
Education of members (in years)							
No education	19.7	3.2	8.1	2.3	9.9	5.6	1.4
Less than 5 years	10.5	1.9	1.9	1.3	2.3	3.6	1.0
5–9 years	7.9	1.2	0.9	1.0	1.1	3.5	1.0
10 years or more	4.7	0.6	0.3	0.6	0.4	2.5	0.8
Wealth Quintile							
Poorest	12.3	2.1	2.3	1.7	2.5	4.4	1.2
Poorer	11.0	1.9	2.0	1.5	2.2	4.1	1.2
Middle	10.0	1.7	1.9	1.2	2.0	3.8	1.2
Richer	8.4	1.4	1.4	0.9	1.7	3.4	1.1
Richest	6.3	0.8	1.1	0.7	1.4	2.8	0.7
Religion							
Hindu	9.5	1.5	1.7	1.2	1.9	3.7	1.0
Muslim	9.6	1.8	1.9	1.2	2.1	3.6	1.1
Christion	9.9	1.6	2.0	1.0	2.1	3.3	1.2
Others	11.0	1.3	1.7	1.3	2.1	4.6	1.7
Caste							
SC/ST	9.9	1.6	1.8	1.4	1.9	3.7	1.1
OBC	9.7	1.6	1.8	1.1	2.0	3.8	1.1
Others	8.9	1.4	1.7	1.1	1.9	3.6	1.0
Residence							
Urban	8.3	1.3	1.6	0.9	1.8	3.4	0.9
Rural	10.1	1.7	1.9	1.3	2.0	3.8	1.1
Education level of cluster							
Low	10.0	1.6	1.9	1.3	2.0	3.9	1.1
Medium	10.4	1.7	1.9	1.3	2.0	4.0	1.2
High	8.4	1.4	1.4	0.9	1.9	3.3	0.9
Wealth status of cluster							
Low	9.9	1.7	2.0	1.4	1.9	3.7	1.0
Medium	10.4	1.6	1.9	1.4	2.0	4.0	1.2
High	8.6	1.4	1.5	0.9	1.9	3.4	1.0
Education level of District							
Low	9.6	1.5	1.8	1.3	1.9	3.7	0.9
Medium	9.7	1.5	1.8	1.3	1.9	3.8	1.2
High	9.4	1.6	1.6	1.0	2.0	3.6	1.1
Wealth status of District							
Low	9.5	1.6	1.9	1.3	1.9	3.6	0.9
Medium	10.0	1.6	1.7	1.3	1.9	3.8	1.2
High	9.3	1.5	1.6	1.0	2.0	3.7	1.1
Education level of State							
Low	9.3	1.5	1.8	1.3	1.9	3.8	0.9
Medium	9.5	1.4	1.7	1.2	1.8	3.6	1.3
High	10.3	2.0	1.8	1.0	2.4	3.7	1.1
Wealth status of State							
Low	9.8	1.6	1.9	1.3	1.9	3.8	0.9
Medium	9.2	1.3	1.6	1.2	1.8	3.6	1.2
High	10.3	2.0	1.8	1.0	2.4	3.7	1.1
Total	9.5	1.6	1.8	1.2	1.9	3.7	1.1

**Fig. 2:**
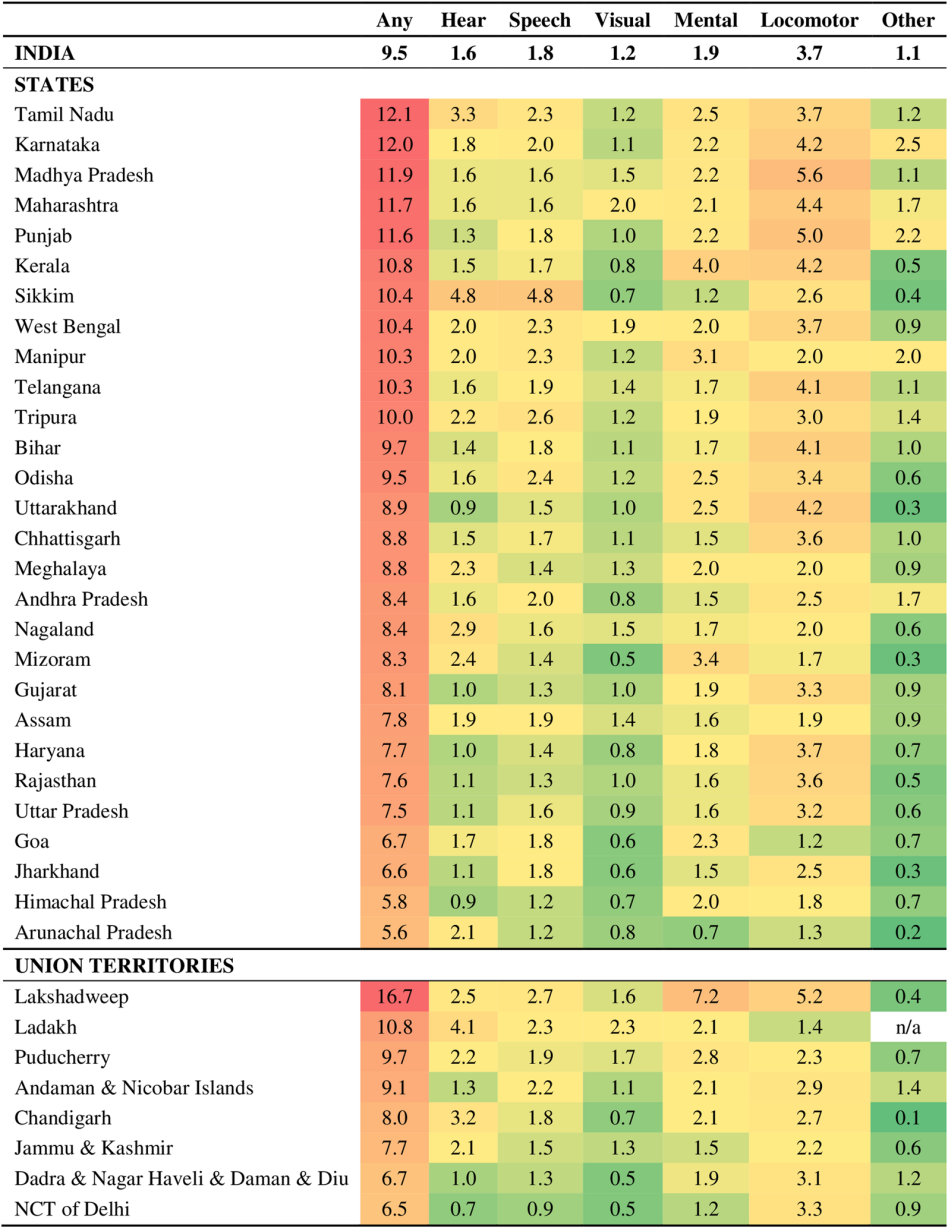
State/UT-specific age-sex adjusted prevalence of disability by its type (per 1000 population) in India, NFHS 2019–21

Figure 2 presents the age-sex-adjusted disabilities (per 1000) across the states/UTs of India. Higher disability rates were found among the participants in southern states of India, such as Tamil Nadu and Karnataka. Central and western states like Madhya Pradesh and Maharashtra and northern states/UTs like Punjab and Ladakh also show higher disability rates. The largest contributor in all these states/UTs was locomotor disability. Moreover, Tamil Nadu shows higher hearing disability too. Followed by these states, eastern states like West Bengal, Bihar, and Odisha, along with north-eastern states like Manipur, Tripura lay in the second highest category of having any disability. Surprisingly, amid these eastern states, Jharkhand shows a lower disability among participants. Mental disability was highest in Kerala. Participants in states like Odisha and Tripura show a higher incidence of speech disabilities.

### Socioeconomic variations in disabilities

**Fig. 3:**
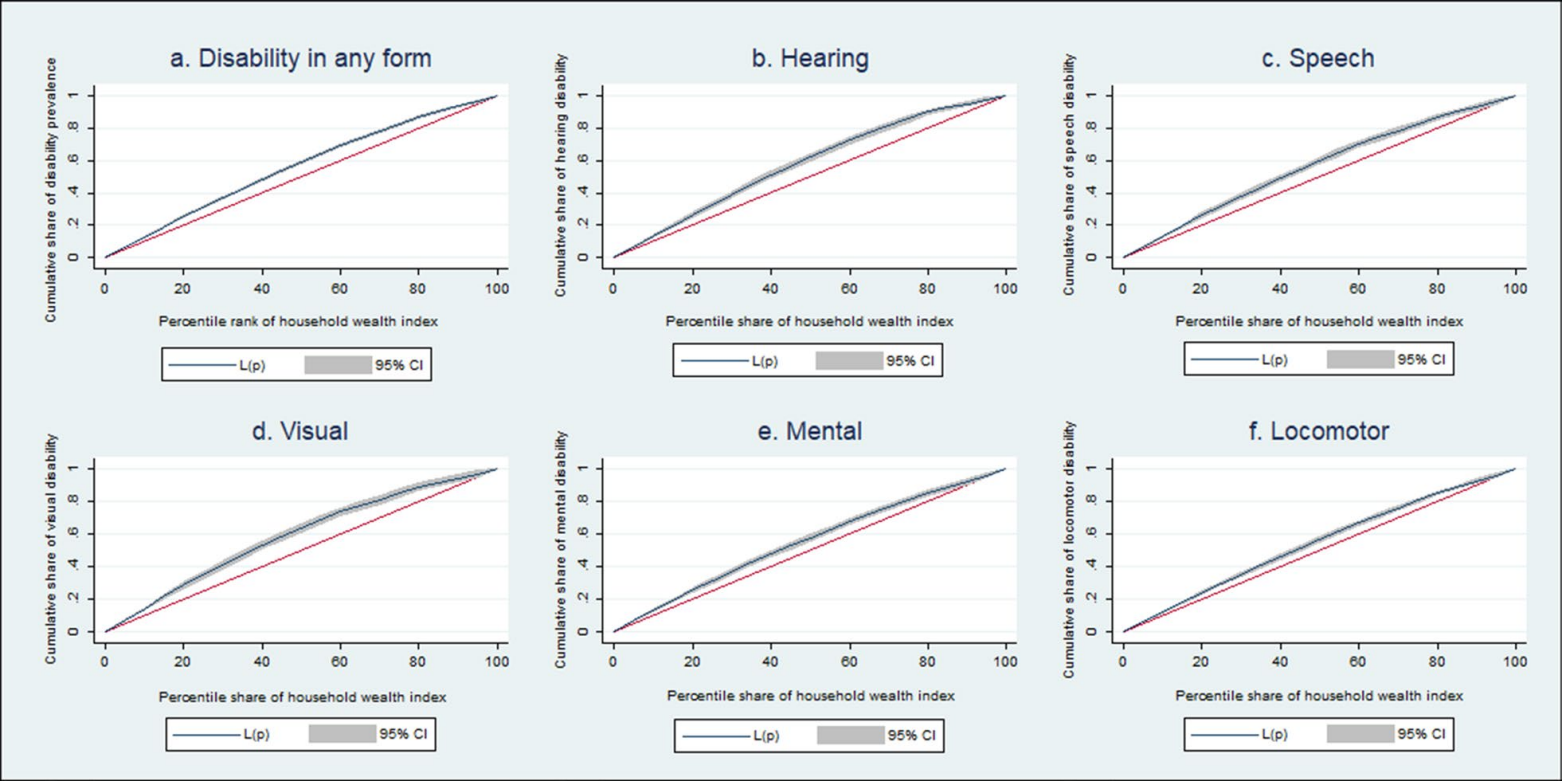
Concentration curves for different types of disability by household wealth status in India, NFHS 2019–21. **a** Disability in any form **b** Hearing **c** Speech **d** Visual **e** Mental **f** Locomotor

The magnitude of relative socioeconomic variations in disabilities is presented in Additional file 1: Table S5. The negative value of the age-sex-adjusted concentration index confirms the presence of socioeconomic inequalities in any form of disability (EI = −0.0023) and with EI = −0.0007 for locomotor disability. For all types of disabilities, the concentration curve lies above the line of equality, indicating that disabilities were disproportionately higher in the poorest quintile household (Fig. 3a–f). Further, the sensitivity analyses confirm the education-related inequality in disability among 18 + individuals (Additional file 1: Table S6). The estimates of education-related inequality were negative with EI = −0.0046 in any form of disability and highest among speech (EI = −0.0015) and mental (EI = −0.002) disabilities. The concentration curve confirms that disability was concentrated among illiterate 18 + individuals Additional file 1: Figure S7a–f).

Additional file 1: Figure S8 presents the adjusted coefficients obtained from the two-level random intercept logit model. After controlling covariates, children aged 0–9 years were less likely to have any disability than older adults in the 60 + age group. However, speech and mental disabilities were higher in 10–29 aged individuals compared to 60 + elderly. Females were less likely to have any disability than their male counterparts. The socioeconomic gradient in any disability remained strong. For instance, the richest wealth quintile households were 0.57 times lesser likely to have any disability compared to those in the poorest wealth quintile (Additional file 1: Table S9). This decrement remains consistent for all the disability types except speech and mental disabilities. Ten years or more of education significantly reduces the chances of disability. Rural residents were higher likely to have speech and mental disabilities.

### The relative importance of geographic levels

**Fig. 4:**
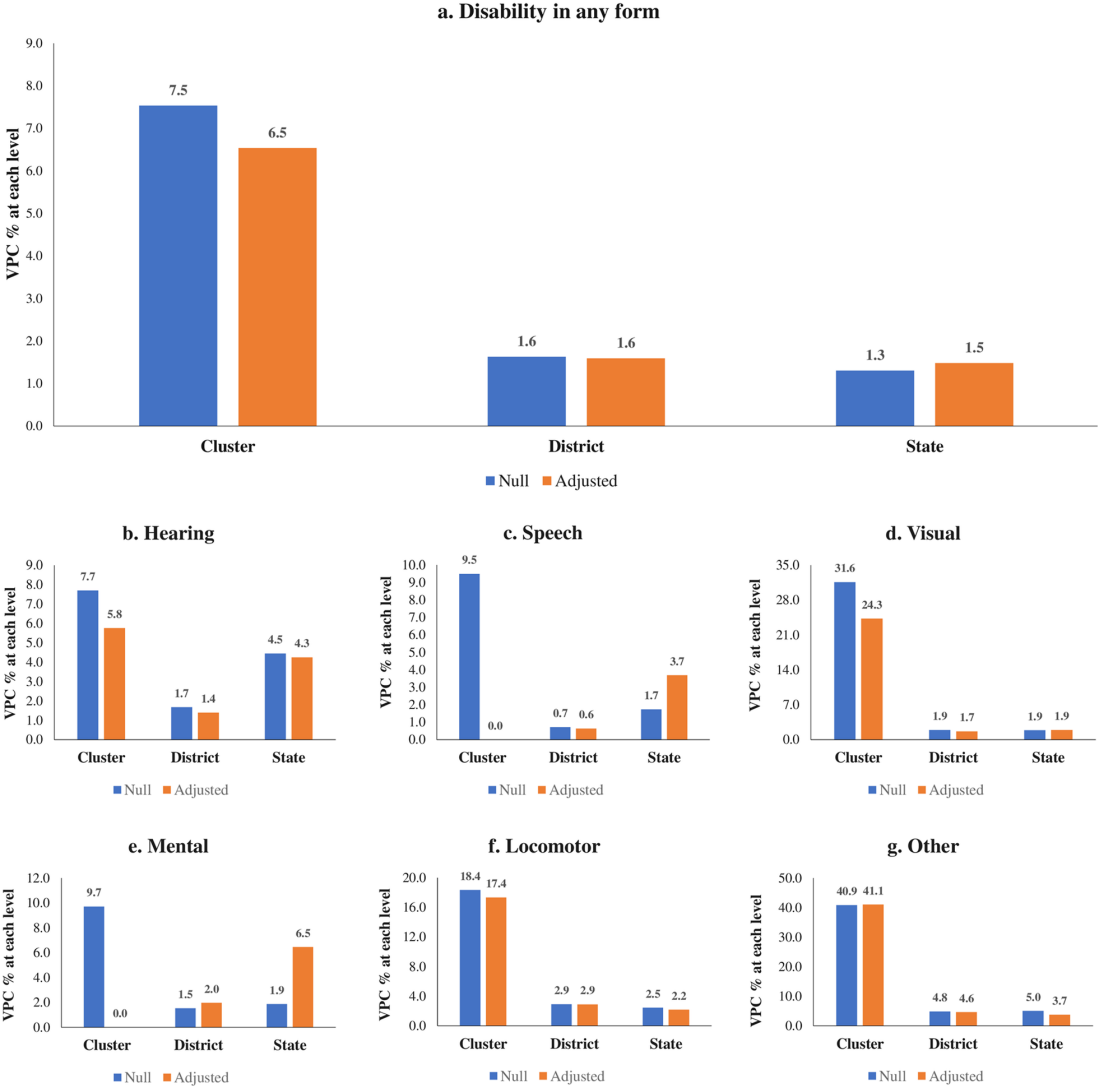
Percentage variation in disability attributable to cluster, district and state levels in null and adjusted models in India, NFHS 2019–21. **a** Disability in any form **b** Hearing **c** Speech **d** Visual **e** Mental **f** Locomotor **g** Other

The variance partitioning estimates from the four-level null model indicate that the largest share of geographical variation in all types of disability is attributed to the clusters (Fig. 4a and b–g). Even after adjusting for the covariates, clusters share the largest source of geographic variation for the following outcomes: (a) any disability (6.5%); (b) hearing (5.8%); (c) visual (24.3%); (d) locomotor (17.4%); (e) other (41.1%). In the adjusted model, states/UTs accounts for the highest variation in speech (3.7%) and mental (6.5%) disabilities, where the variation at the cluster level becomes negligible. Districts were the second largest source of geographic variation for any disability (1.6%) and locomotor disability (2.9%).

### Sensitivity analyses

Table 2 presents the sensitivity analyses showing changes in the variance estimates and proportion of the total variation attributable to the higher levels from four levels to two-level fully adjusted model specification. The results of the two-level model show clusters to be the largest source of geographic variation in all types of disability. For instance, a model with individuals (level-1) nested within clusters only (level-2) shows a between-cluster variation of 10.89% in any disability condition. They are followed by between-district and between-state variations such as 3.55% and 1.96% respectively. The cluster level variations were nearly six times greater than what was found in the four-level model (VPC% at two-level: 5.76; VPC% at four-level: 30.41). The negligible variation between clusters in speech and mental disabilities were substantially increased to 19.85% and 22.06%, respectively. When individuals were assumed to be nested within districts only, there were two to six times greater variations in different types of disability compared to four-level models. However, the between-state variations from four-level to two-level model specifications showed a less dramatic increase.

**Table 2:** Sensitivity analyses showing variance estimates and proportion of total variation in disabilities attributable to cluster, district, and state levels in adjusted four-level and two-level models in India, NFHS 2019–21

Type of disability	Cluster		District		State	
	Variance estimate	% Variance attributable	Variance estimate	% Variance attributable	Variance estimate	% Variance attributable
Any						
Four level model	0.238	6.54	0.058	1.59	0.054	1.48
Two level models	0.402	10.89	0.121	3.55	0.066	1.96
Hearing						
Four level model	0.214	5.76	0.052	1.40	0.158	4.25
Two level models	1.438	30.41	0.196	5.62	0.162	4.68
Speech						
Four level model	0.000	0.00	0.022	0.64	0.127	3.69
Two level models	0.815	19.85	0.125	3.67	0.131	3.84
Visual						
Four level model	1.108	24.29	0.076	1.67	0.088	1.93
Two level models	1.545	31.95	0.211	6.02	0.095	2.81
Mental						
Four level model	0.000	0.00	0.071	1.98	0.232	6.46
Two level models	0.931	22.06	0.254	7.18	0.263	7.39
Locomotor						
Four level model	0.736	17.35	0.123	2.90	0.092	2.17
Two level models	0.654	16.58	0.190	5.47	0.104	3.07
Other						
Four level model	2.672	41.07	0.302	4.64	0.242	3.72
Two level models	2.782	45.82	0.566	14.69	0.276	7.74

Both four-level and two-level models are fully adjusted

### District-level precision weighted estimates and correlation with small area variations

**Fig. 5:**
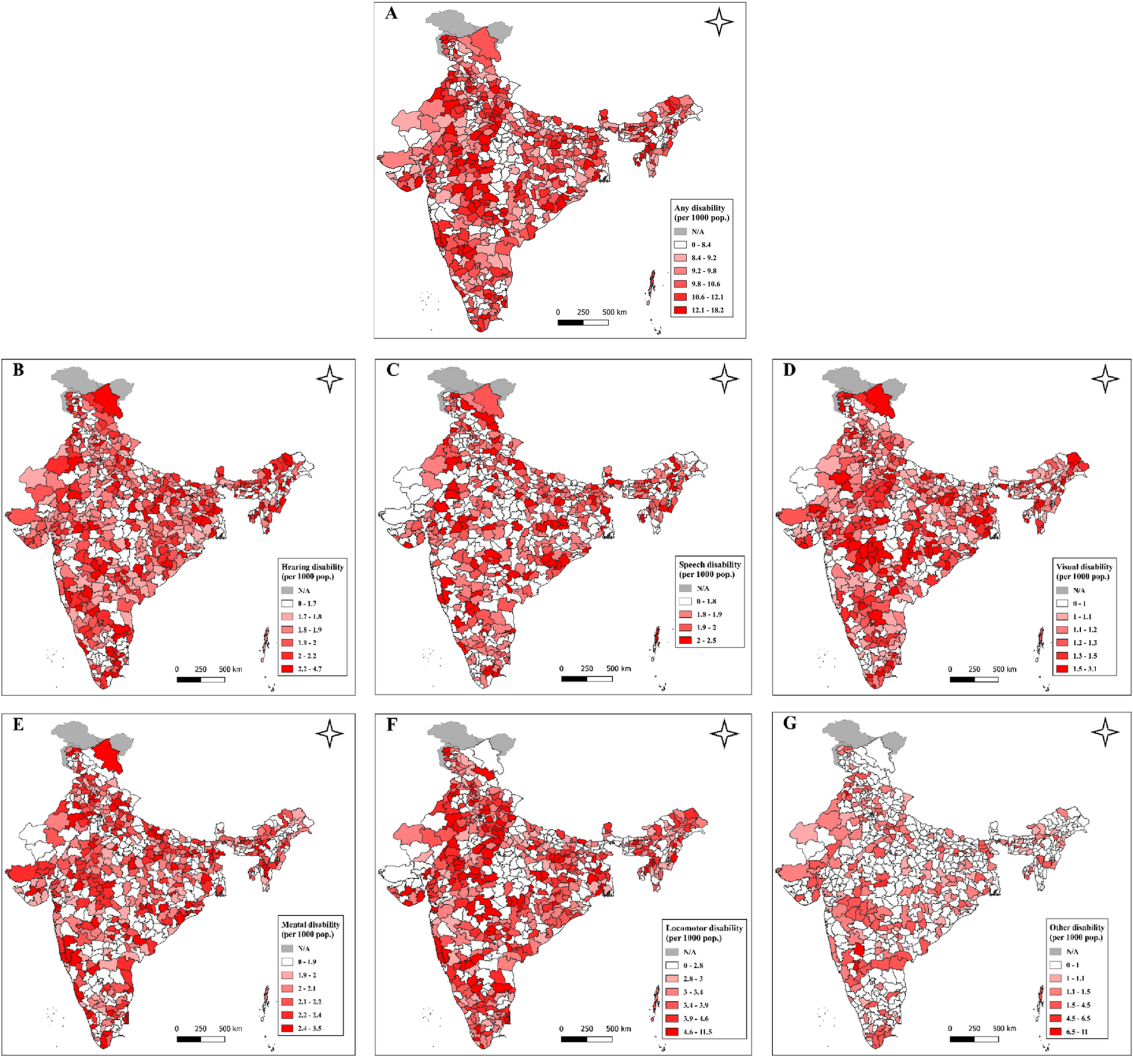
Maps showing geographic distribution of disability rates (per 1000 population) across 707 districts in India, NFHS 2019–21. **a** Disability in any form **b** Hearing **c** Speech **d** Visual **e** Mental **f** Locomotor **g** Other. All maps are author’s own creation in QGIS 3.28 software using base map of https://globalsolaratlas.info/download/india

The spatial representation of precision-weighted estimates shows significant variation in all types of disability across the districts. The highest disability rate (per 1000 population), ranging from 12.1 to 18.2, was found mostly in parts of Madhya Pradesh, Maharashtra, Karnataka, Tamil Nadu, Telangana, and Punjab (Fig. 5a). Hearing disability was more concentrated across Ladakh, Uttarakhand, Maharashtra, Tamil Nadu, and a few north-eastern states like Nagaland, Arunachal Pradesh (Fig. 5b–g). Delhi district's National Capital Territory (NCT) shows the highest visual disability rate (3.1 persons per 1000 population). Speech disability was scattered across the districts of all regions. Mental disability was higher in central, western, and southern parts like Madhya Pradesh, Maharashtra, Karnataka, and Tamil Nadu. Kozhikode district of Kerela shows the highest mental disability rate in India (3.5 persons per 1000 population). Districts like Rajgarh, Datia, Shajapur, Ashoknagar, Mandsaur, Sehore, and Bhind show higher locomotor disability rates ranging from 11.5 to 7.6. In the midst of these districts, Jehanabad district of Bihar (8.8 per 1000) and Kurukshetra district of Haryana (7.9 per 1000) experiences higher locomotor disability.

**Fig. 6:**
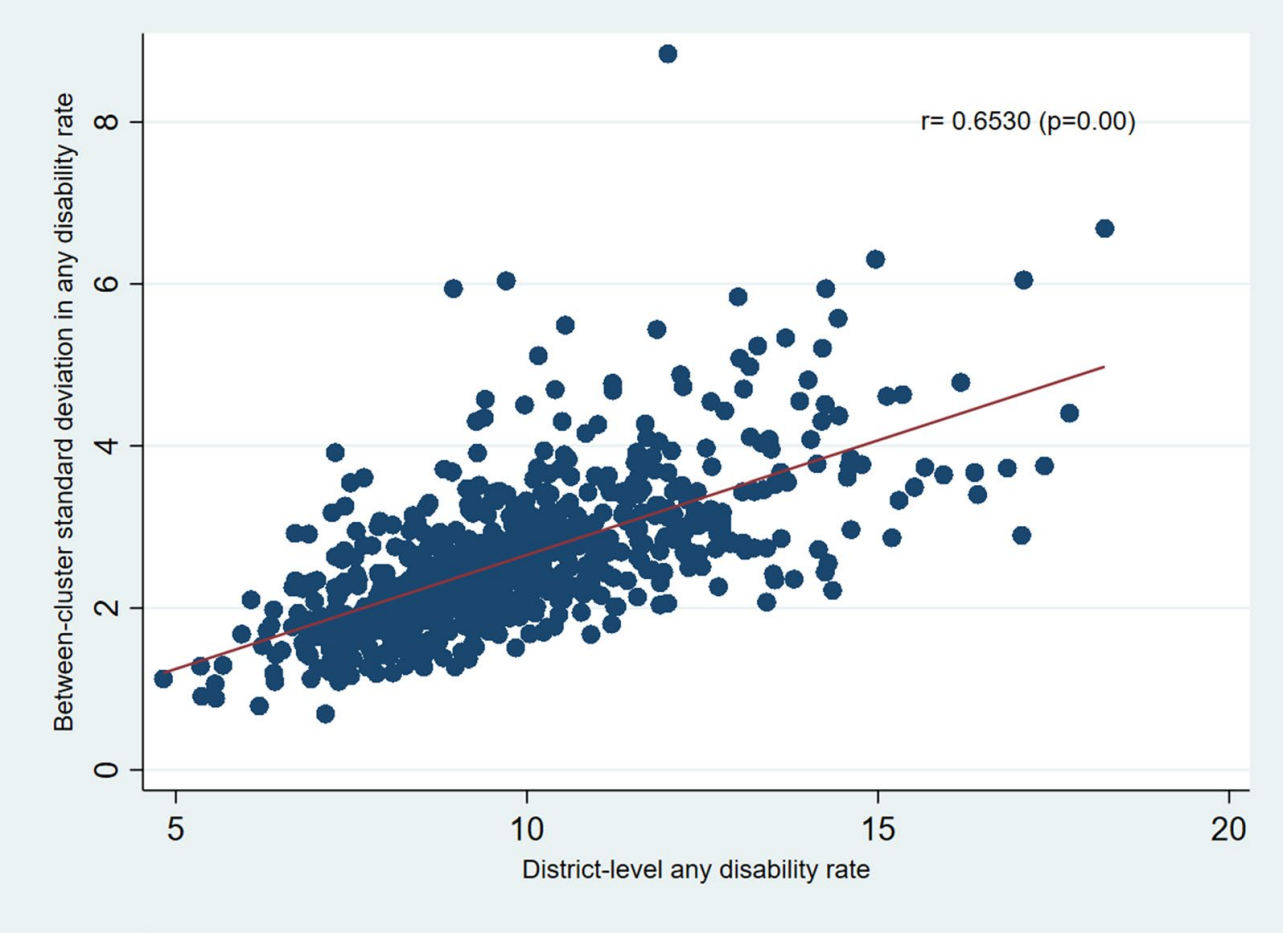
Correlation between the district-level rates and within-district or between-cluster standard deviation of disability in India, NFHS 2019–21

Further, we computed the between-cluster standard deviations (SDs) for each outcome by district which were used to show correlations with the between-district disability rates. Results from Fig. 6 show a positive correlation between the district rates and cluster SDs for any disability condition (r = 0.65, p < 0.001). We also found a significant positive correlation for the district rates and cluster SDs of the hearing (r = 0.75, p < 0.001), speech (r = 0.70, p < 0.001), visual (r = 0.41, p < 0.001), mental (r = 0.73, p < 0.001) and locomotor disabilities (r = 0.54, p < 0.001) (Additional file 1: Figure S10a-f).

## Discussion

Disability, a multidimensional concept, has always been discussed as a public health concern in India [2, 10, 48]. Though previous studies provided the estimates and patterns of disability, limited studies show socioeconomic and geographic variations in disabilities using a nationally representative sample of India. Socioeconomic inequality or variation, which lies at the root of various health inequalities, is the biggest hindrance to India’s development process. The present study confirms the presence of higher socioeconomic inequalities in disability prevalence by estimating a higher concentration of all types of disabilities in the poorest wealth quintile household and illiterate individuals. Further, we found significant geographic variations in disabilities existed between and within India’s clusters, districts, and states. Following are the several notable findings of the study:

First, we found that about 10 per 1000 Indian population had at least one disability. Among the disabled population, locomotor disability was reported to be the highest. The plausible reason for such higher rates may be the easier identification of locomotor disabilities than others—moreover, the definition of disability matters here [49]. For instance, in the case of visual disability, some surveys consider blurred vision a disability, but without a proper process, it may be misclassified [10]. Nearly all types of disability rates were higher in the 60 + age group, except speech and mental disability which were higher in growing ages of individuals (10–29 years). Consistent with an Indian study, southern and central states like Kerala, Karnataka, Tamil Nadu, Maharashtra, and Madhya Pradesh showed the highest disability rates [11].

Second, the Erreygers concentration index revealed that all types of disabilities were common among most disadvantaged or lower socioeconomic groups. These findings are consistent with those from developing nations, where disability risks were higher in poorer households [30, 50]. Such evidence may be due to the bidirectional relationship between socioeconomic status and disability [51]. For instance, negative societal attitudes, marginalization, and stigma may exclude disabled persons from proper education, employment, and health services leading them to poorer socioeconomic status. On the other hand, individuals from poorer households have a higher risk of disability due to poor health (i.e., constant disease risks), lack of adequate health intervention (i.e., untimely receiving services like immunization), poor living conditions (i.e., lack of healthy sanitation and drinking practice), and higher risk of injuries (i.e., by unsafe work or during disaster situation) [51]. Although poor socioeconomic status is highly associated with disability, those living in the richest household are also not spared from this risk [30]. For instance, a study from Cambodia shows that disabilities due to road accident injuries were higher among wealthy households with greater ownership of vehicles [52]. However, with the growing availability of resources, the wealthy can also recover from the day-to-day difficulties of disability. Different medical technologies can help individuals without legs to walk, without eyes to see the world again, heal from speech disability, etc. But all these health interventions cost high. So, even if available, individuals from poorer socioeconomic status cannot afford these services and are forced to live low-quality lives. Besides wealth-related variations, the present study found vast education-related inequalities in disabilities. For instance, the concentration of disabilities were higher in illiterate 18 + individuals. These might be due to the vicious cycle of health and education where disabled people are denied educational opportunities, and those illiterate may contact disability much higher due to unawareness [53, 54].

Third, clusters were found to be the largest source of geographic variation for any disability condition, along with hearing, visual, and locomotor. Furthermore, states share the largest source of geographic variation in speech and mental disability. The sensitivity analyses comparing four-level and two-level models confirm that clusters have the highest share of variance in disabilities. However, a dramatic variance increase in the two-level model suggests that considering variations at single or two levels may be highly misleading for a study. Fourth, districts with the highest disability rates were clustered in Madhya Pradesh, Maharashtra, Karnataka, Tamil Nadu, Telangana, and Punjab. It was worth noting that both high-income states (i.e., Tamil Nadu) and low-income states (i.e., Madhya Pradesh) were among the most disabled population. The spatial results show wide geographic variations in the risk of different types of disability across the districts of India. For instance, while hearing and speech disabilities were highly concentrated in parts of Tamil Nadu and Madhya Pradesh, respectively, visual disability was highest in the NCT of Delhi. Mental disability was highest in Kozhikode district of Kerala, and different districts of Madhya Pradesh topped the list of locomotor disability in India. Fifth, we found positive correlations between the district rates and cluster SDs for disabilities. This implies that districts with higher disability rates have a larger degree of cluster-level variations.

Though the disability estimates were unclear in the Indian context, the present study takes advantage of recent data to explore India's socioeconomic and geographic variations in disabilities. Higher socioeconomic inequalities in all types of disabilities in the present study indicated the widening health disparities in the Indian population. The present study provides a broad picture for policymakers to focus on the poorer socioeconomic status population. Moreover, with a large concentration of disabilities in the illiterate, the present study highlights the educational limitations of having a disability. Although past evidence had shown the spatial distribution of disabilities across states and NSS regions [32, 33]. The larger share of variation attributable to small areas like clusters indicates the importance of targeting lower geographic levels for studies and policy implementation. The sensitivity analyses further confirm the relevance of exploring small area variations considering all four levels simultaneously. Our results identify the cluster-level factors (like wealth status and education) explaining small area variation in disabilities. These factors must be addressed while implementing policies at smaller geographic levels. Further, wide geographic variations across the districts indicated that the high-income and low-income states should be prioritized for intervention.

Despite the advantages in the study, there are limitations as well. Firstly, accessing data on disability is challenging due to limited surveys covering this multidimensional concept and definitional variations across countries. For example, HIV/AIDS is considered a disability in South Africa, but this is not the case in countries with low HIV rates, such as India. Consequently, the Short Set Questionnaire based on the World Health Organization’s International Classification of Function, Disability, and Health is widely accepted for standardization. However, the existence of varying definitions leading to inconsistent estimates is not only a global issue but is also evident at the national level. Nationally, significant differences in disability estimates between self-recalled and other sources arise from subjective definitions of disability.

In India, while the Census of India serves as the largest source of disability estimates, the National Sample Survey is the primary survey dedicated to the disabled population. However, disability estimates on a global scale remain insufficient. The inclusion of disability in the National Family Health Survey (NFHS), a component of the Demographic Health Survey, marked a significant step in conducting global health research. Although the disability definitions in NFHS generally align with the patterns of Census and NSS, discrepancies in estimates underscore the need for proper standardization of disability measurements. This should encompass a range of activity limitations, participation restrictions, and impairments, with an emphasis on improving accuracy by questioning each household member about their condition. The practice of answering disability questions by the household head or representative, instead of each member, often results in misreporting, particularly among the wealthier section of society. This is attributed to stigma, negative societal behavior, and the fear of losing power [51]. Consequently, surveys in India encounter numerous challenges in obtaining accurate disability estimates. Despite these challenges, the simplified questions posed to a large, representative set in NFHS can yield remarkable insights into the disabled population of India. Second, although socioeconomic status and disability are highly linked, we could only capture the cross-sectional association. Thus, causality can’t be inferred. Third, due to the data availability, we could only capture the disabilities related to physical conditions. There is a need for future research and surveys to focus on environmental and personal factors too. Fourth, the present study did not consider health variables as we aim to show variations in disability status. Thus, future research should focus on the association between disease and disability in India.

## Conclusions

Along with the growing concern about disability conditions in India, the present study presses the need to focus on the emerging issue of disability variations across socioeconomic groups and geographic locations in India. Education about the cause and consequences of disability should be prioritized, especially among the poorer socioeconomic groups and the illiterate. Educating disadvantaged groups can reduce delays in healthcare-seeking behavior and lack of knowledge or awareness about early symptoms of diseases, which are the prominent reasons for disability onset. The importance of cluster in the present study suggests the need to consider small area variation for implementing several policy-relevant implications. For instance, within-districts or between-districts-targeted policies are required as variability may be due to variations in the progress of health programs or vulnerability status. Moreover, the incidence of underreporting may cover the actual disability situation of districts and states in India. So, policymakers should enforce disability awareness programs and disability-access facilities in education, employment, transportation, health sectors, public toilets, etc., especially at smaller geographic levels, as this can become a first milestone for the communities to understand disability. There is also a need for better-quality monitoring data to record the presence of these disability-accessible services to eliminate discrimination across vulnerable groups.

### Supplementary Information

**Additional file 1: Figure S1.** Schematic representation of the four-level hierarchical structure of the final analytic sample, NFHS 2019–21, India. **Table S2.** Distribution of number of districts, clusters, individuals, and disabled individuals, within 36 Indian states/union territories, NFHS 2019–21, India. **Table S3.** Description of various types of disability considered in NFHS 2019–21. **Table S4.** Sample distribution of participant’s characteristics in NFHS 2019–21, India. **Table S5.** Age-sex adjusted wealth-related inequalities in the prevalence of disability by its type in India, NFHS 2019–21. **Table S6.** Age-sex adjusted education-related inequalities in the prevalence of disability by its type in 18 + individuals of India, NFHS 2019–21. **Figure S7. a-f:** Concentration curves for different types of disability by education of 18 + individuals in India, NFHS 2019–21. (a.) Disability in any form (b.) Hearing (c.) Speech (d.) Visual (e.) Mental (f.) Locomotor. **Figure S8.** Multilevel logistic regression model showing association of covariates on disability by its type in India, NFHS 2019–21. **Table S9.** Four-level random intercept logit model showing risk factors associated with disability across its type in India, NFHS 2019–21. **Figure S10. a-f:** Correlation between the district-level rates and within-district or between-cluster standard deviation of disability by its type in India, NFHS 2019–21. (a.) Hearing (b.) Speech (c.) Visual (d.) Mental (e.) Locomotor (f.) Other.

## Data Availability

All the data is available in the public domain at https://dhsprogram.com/data/dataset/India_Standard-DHS_2020.cfm?flag=0.

## Abbreviations

CI: Relative concentration index
EI: Erreygers concentration index
IGLS: Iterated generalized least square
NCT: National capital territory
NFHS: National Family Health Survey
NSS: National Sample Survey
OBC: Other backward caste
PCA: Principal component analysis
SC: Scheduled caste
SD: Standard deviation
ST: Scheduled tribe
UT: Union territory
VPC: Variance partitioning coefficient
WHO: World Health Organization
