# Supplementary material for: Socioeconomic and geographic variations of disabilities in India: evidence from the National Family Health Survey, 2019–21
Source: Int J Health Geogr. 2024 Feb 18;23:4. doi: 10.1186/s12942-024-00363-w (PMC10874552; doi:10.1186/s12942-024-00363-w)

**S1 Fig:** Schematic representation of the four-level hierarchical structure of the final analytic sample, NFHS 2019-21, India.

**LEVEL 4:** States/UTs (n= 36)

Including only de jure population (n=27,95,894) and excluding missing values of covariates (n=1,923)

Total number of individuals interviewed (n=28,43,917) from households (n=636,699)

**LEVEL 1:** Individuals (n= 27,93,971)

**LEVEL 2:** Clusters (n= 30,170)

**LEVEL 3:** Districts (n= 707)

**S2 Table:** Distribution of number of districts, clusters, individuals, and disabled individuals, within 36 Indian states/union territories, NFHS 2019-21, India.

| **STATES** | **# Districts** | **# Clusters** | **# Individuals** | **# Disabled** |
| --- | --- | --- | --- | --- |
| Andhra Pradesh | 13 | 546 | 40,170 | 353 |
| Arunachal Pradesh | 20 | 858 | 70,332 | 404 |
| Assam | 33 | 1,386 | 1,31,247 | 956 |
| Bihar | 38 | 1,710 | 1,72,606 | 1,579 |
| Chhattisgarh | 27 | 1,134 | 1,07,309 | 961 |
| Goa | 2 | 84 | 7,224 | 48 |
| Gujarat | 33 | 1,386 | 1,32,483 | 1,080 |
| Haryana | 22 | 877 | 88,982 | 733 |
| Himachal Pradesh | 12 | 504 | 40,761 | 256 |
| Jharkhand | 24 | 1,080 | 1,02,641 | 680 |
| Karnataka | 30 | 1,260 | 1,14,535 | 1,575 |
| Kerala | 14 | 588 | 45,716 | 534 |
| Madhya Pradesh | 51 | 2,131 | 1,99,794 | 2,467 |
| Maharashtra | 36 | 1,510 | 1,35,686 | 1,791 |
| Manipur | 9 | 378 | 33,340 | 335 |
| Meghalaya | 11 | 462 | 50,554 | 406 |
| Mizoram | 8 | 336 | 29,198 | 257 |
| Nagaland | 11 | 461 | 35,286 | 358 |
| Odisha | 30 | 1,260 | 1,07,200 | 1,075 |
| Punjab | 22 | 915 | 87,576 | 1,109 |
| Rajasthan | 33 | 1,485 | 1,60,956 | 1,211 |
| Sikkim | 4 | 168 | 11,769 | 137 |
| Tamil Nadu | 32 | 1,344 | 94,359 | 1,256 |
| Telangana | 31 | 1,302 | 97,506 | 1,090 |
| Tripura | 8 | 336 | 26,647 | 281 |
| Uttar Pradesh | 75 | 3,358 | 3,65,893 | 2,672 |
| Uttarakhand | 13 | 582 | 50,931 | 465 |
| West Bengal | 20 | 840 | 72,941 | 818 |
| **UNION TERRITORIES** |  |  |  |  |
| A&N Islands | 3 | 126 | 9,367 | 97 |
| Chandigarh | 1 | 41 | 3,347 | 27 |
| Dadra & Nagar Haveli & Daman & Diu | 3 | 126 | 10,268 | 95 |
| Jammu & Kashmir | 20 | 840 | 88,842 | 710 |
| Ladakh | 2 | 84 | 8,485 | 93 |
| Lakshadweep | 1 | 42 | 4,258 | 67 |
| NCT of Delhi | 11 | 462 | 42,816 | 287 |
| Puducherry | 4 | 168 | 12,946 | 131 |
| **INDIA** | **707** | **30,170** | **27,93,971** | **26,394** |

**S3 Table:** Description of various types of disability considered in NFHS 2019-21.

| **Disability Type** | **Description** |
| --- | --- |
| Hearing | Persons who cannot hear at all and those having difficulty in hearing day-to-day conversational speech with or without a hearing aid will be classified as having a hearing disability. The presence of a hearing disability will be decided taking into account the functioning of both the ears. Persons having a problem in only one ear will not be considered as having a hearing disability. |
| Speech | A person will be classified as having a speech disability if she/he cannot speak at all or she/he is unable to speak normally due to a speech disorder. Persons who speak in single words will be treated as having a speech disability. Persons having articulation defects and those who stammer will also be coded as having a speech disability. However, persons who stammer but whose speech is comprehensible will not be classified as having a speech disability. The presence of a speech disability can be assessed only after the age of three years in children. |
| Visual | Persons with no perception of light and those who perceive light but cannot see properly on account of low and/or blurred vision will be treated as suffering from a visual disability. Persons who suffer from low vision even after taking corrective measures (using spectacles, contact lenses, etc.), will also be considered as being visually disabled. Persons who do not have difficulty in seeing after taking corrective measures will not be treated as disabled. Persons with proper vision in one eye (one eyed persons) will not be treated as visually disabled. |
| Mental | Persons who lack understanding appropriate to their age and have difficulty in carrying out the activities of a daily routine like others of similar age such as communication (speech), self-care (brushing of teeth, wearing clothes, taking a bath, taking food, personal hygiene, etc.) will be treated as mentally disabled. Those having problem in communicating and understanding verbal and nonverbal messages will be treated as mentally disabled. The category of mentally disabled include both persons with mental retardation and those who are mentally ill (one need not try to distinguish between them). Please note that persons, who show signs of mental fatigue, lack of understanding, and being dependent on others for their daily routine on account of being old, will not be considered as mentally disabled. |
| Locomotor | Persons who are deprived of the use of a limb (those who do not have both their legs and their hands or those who do not have the use of both of their legs or both of their hands) or persons who are paralyzed and are unable to move at all will be treated as having locomotor disability. Persons with loss or absence of whole or part of hand or leg and persons suffering from inactivity of whole or part of body due to amputation, paralysis, deformity (including hunch back, deformed spine, etc.) or dysfunction of limbs or joints which affected her/his 'normal' ability to move (with or without aid) self or objects will also be considered as disabled. Persons using artificial limbs will be treated as having locomotor disability. Persons with temporary disability on the date of the survey like stiff neck; back injury; fracture of hand(s), leg(s), etc. will not be treated as disabled. |
| Other | Any other type of disability not covered above |

**Note: These descriptions can be found on NFHS site “**[NFHS-5 Interviewer Manual_Eng.pdf (rchiips.org)](http://rchiips.org/NFHS/NFHS5/manuals/NFHS-5%20Interviewer%20Manual_Eng.pdf)”

**S4 Table:** Sample distribution of participant’s characteristics in NFHS 2019-21, India.

| **Participant Characteristics** | **N** | **%** |
| --- | --- | --- |
| **Age of household members** |  |  |
| 0-9 | 4,85,598 | 17.1 |
| 10-19 | 5,18,065 | 18.4 |
| 20-29 | 4,65,865 | 16.7 |
| 30-39 | 4,01,587 | 14.3 |
| 40-49 | 3,34,494 | 12.1 |
| 50-59 | 2,68,375 | 9.6 |
| 60+ | 3,19,987 | 11.8 |
| **Sex of household members** |  |  |
| Male | 13,91,597 | 49.7 |
| Female | 14,02,374 | 50.3 |
| **Education of members (in years)** |  |  |
| No Education | 8,80,112 | 30.7 |
| Less than 5 years | 3,60,748 | 12.8 |
| 5-9 years | 8,00,681 | 28.2 |
| 10 years or more | 7,52,430 | 28.4 |
| **Wealth Quintile** |  |  |
| Poorest | 6,25,672 | 20 |
| Poorer | 6,16,822 | 20 |
| Middle | 5,65,250 | 20 |
| Richer | 5,15,500 | 20 |
| Richest | 4,70,727 | 20 |
| **Religion** |  |  |
| Hindu | 20,97,509 | 81.0 |
| Muslim | 3,57,315 | 13.7 |
| Christion | 2,01,259 | 2.4 |
| Others | 1,37,888 | 2.9 |
| **Caste** |  |  |
| SC/ST | 10,73,548 | 31.4 |
| OBC | 10,40,008 | 42.0 |
| Others | 6,80,415 | 26.6 |
| **Residence** |  |  |
| Urban | 6,77,176 | 31.8 |
| Rural | 21,16,795 | 68.2 |
| **Education level of Cluster** |  |  |
| Low | 9,84,073 | 32.7 |
| Medium | 9,39,860 | 33.6 |
| High | 8,70,038 | 33.7 |
| **Wealth status of Cluster** |  |  |
| Low | 9,56,450 | 28.9 |
| Medium | 9,42,443 | 33.8 |
| High | 8,95,078 | 37.3 |
| **Education level of District** |  |  |
| Low | 9,83,795 | 31.0 |
| Medium | 9,49,328 | 34.6 |
| High | 8,60,848 | 34.4 |
| **Wealth status of District** |  |  |
| Low | 9,63,064 | 29.1 |
| Medium | 9,43,199 | 32.1 |
| High | 8,87,708 | 38.9 |
| **Education level of State** |  |  |
| Low | 12,99,664 | 44.9 |
| Medium | 9,68,995 | 39.1 |
| High | 5,25,312 | 16.0 |
| **Wealth status of State** |  |  |
| Low | 11,09,897 | 34.4 |
| Medium | 11,56,979 | 49.6 |
| High | 5,27,095 | 16.0 |
| **Total** | **27,93,971** | **100** |

**S5 Table:** Age-sex adjusted wealth-related inequalities in the prevalence of disability by its type in India, NFHS 2019-21.

| **Types of disability** | **Erreygers Concentration Index** | **95% Confidence Interval** |
| --- | --- | --- |
| **Any** | -0.0023* | -0.0025, -0.0021 |
| **Hearing** | -0.0005* | -0.0006, -0.0004 |
| **Speech** | -0.0005* | -0.0005, -0.0004 |
| **Visual** | -0.0004* | -0.0005, -0.0004 |
| **Mental** | -0.0004* | -0.0005, -0.0003 |
| **Locomotor** | -0.0007* | -0.0008, -0.0005 |
| **Other** | -0.0002* | -0.0002, -0.0001 |

**S6 Table:** Age-sex adjusted education-related inequalities in the prevalence of disability by its type in 18+ individuals of India, NFHS 2019-21.

| **Types of disability** | **Erreygers Concentration Index** | **95% Confidence Interval** |
| --- | --- | --- |
| **Any** | -0.0046* | -0.0049, -0.0044 |
| **Hearing** | -0.0009* | -0.001, -0.0008 |
| **Speech** | -0.0015* | -0.0016, -0.0014 |
| **Visual** | -0.0006* | -0.0007, -0.0005 |
| **Mental** | -0.002* | -0.0021, -0.0019 |
| **Locomotor** | -0.0009* | -0.0011, -0.0008 |
| **Other** | -0.0002* | -0.0003, -0.0001 |

**S7 Figs a-f:** Concentration curves for different types of disability by education of 18+ individuals in India, NFHS 2019-21. (a.) Disability in any form (b.) Hearing (c.) Speech (d.) Visual (e.) Mental (f.) Locomotor.

**S8 Fig:** Multilevel logistic regression model showing association of covariates on disability by its type in India, NFHS 2019-21.

**S9 Table:** Four-level random intercept logit model showing risk factors associated with disability across its type in India, NFHS 2019-21.

| **Variables** | **Type of Disability** | | | | | | | | | | | | |  |
| --- | --- | --- | --- | --- | --- | --- | --- | --- | --- | --- | --- | --- | --- | --- |
|  | **Any** | | **Hearing** | | **Speech** | | **Visual** | | **Mental** | | **Locomotor** | | **Other** | |
|  | **Null** | **Adjusted** | **Null** | **Adjusted** | **Null** | **Adjusted** | **Null** | **Adjusted** | **Null** | **Adjusted** | **Null** | **Adjusted** | **Null** | **Adjusted** |
| **Intercept** | 0.009* | 0.025* | 0.002* | 0.006* | 0.002* | 0.002* | 0.001* | 0.005* | 0.002* | 0.001* | 0.003* | 0.009* | 0.001* | 0.002* |
| **Age of household member** |  |  |  |  |  |  |  |  |  |  |  |  |  |  |
| 60+ (Ref) |  |  |  |  |  |  |  |  |  |  |  |  |  |  |
| 50-59 |  | 0.82* |  | 0.62* |  | 1.21* |  | 0.67* |  | 1.71* |  | 0.76* |  | 0.84* |
| 40-49 |  | 0.94* |  | 0.57* |  | 2.06* |  | 0.61* |  | 3.82* |  | 0.83* |  | 0.75* |
| 30-39 |  | 1.12* |  | 0.64* |  | 3.6* |  | 0.6* |  | 6.67* |  | 0.89* |  | 0.72* |
| 20-29 |  | 1.23* |  | 0.77* |  | 7.46* |  | 0.65* |  | 13.55* |  | 0.71* |  | 0.6* |
| 10-19 |  | 0.92* |  | 0.71* |  | 8.91* |  | 0.51* |  | 11.89* |  | 0.48* |  | 0.48* |
| 0-9 |  | 0.23* |  | 0.15* |  | 0.96 |  | 0.17* |  | 0.71* |  | 0.25* |  | 0.22* |
| **Sex of household member** |  |  |  |  |  |  |  |  |  |  |  |  |  |  |
| Male (Ref) |  |  |  |  |  |  |  |  |  |  |  |  |  |  |
| Female |  | 0.52* |  | 0.67* |  | 0.53* |  | 0.62* |  | 0.48* |  | 0.49* |  | 0.52* |
| **Education (in years)** |  |  |  |  |  |  |  |  |  |  |  |  |  |  |
| No Education (Ref) |  |  |  |  |  |  |  |  |  |  |  |  |  |  |
| Less than 5 years |  | 0.49* |  | 0.51* |  | 0.19* |  | 0.58* |  | 0.16* |  | 0.64* |  | 0.77* |
| 5-9 years |  | 0.36* |  | 0.32* |  | 0.09* |  | 0.42* |  | 0.08* |  | 0.64* |  | 0.81* |
| 10 years or more |  | 0.23* |  | 0.16* |  | 0.03* |  | 0.3* |  | 0.03* |  | 0.49* |  | 0.71* |
| **Wealth Quintile** |  |  |  |  |  |  |  |  |  |  |  |  |  |  |
| Poorest (Ref) |  |  |  |  |  |  |  |  |  |  |  |  |  |  |
| Poorer |  | 0.89* |  | 0.85* |  | 1.05 |  | 0.85* |  | 1.01 |  | 0.89* |  | 0.89 |
| Middle |  | 0.78* |  | 0.73* |  | 1.07 |  | 0.69* |  | 1 |  | 0.79* |  | 0.74* |
| Richer |  | 0.69* |  | 0.6* |  | 1.02 |  | 0.62* |  | 1.01 |  | 0.68* |  | 0.6* |
| Richest |  | 0.57* |  | 0.51* |  | 1.01 |  | 0.48* |  | 0.98 |  | 0.55* |  | 0.43* |
| **Religion** |  |  |  |  |  |  |  |  |  |  |  |  |  |  |
| Hindu (Ref) |  |  |  |  |  |  |  |  |  |  |  |  |  |  |
| Muslim |  | 0.93* |  | 1.03 |  | 0.8* |  | 0.97 |  | 0.87* |  | 0.95 |  | 0.99 |
| Christion |  | 1.12* |  | 1.26* |  | 1.26* |  | 0.96 |  | 1.18* |  | 1.11 |  | 0.99 |
| Others |  | 1.12* |  | 1.02 |  | 0.93 |  | 1.14 |  | 1.05 |  | 1.16* |  | 1.15 |
| **Caste** |  |  |  |  |  |  |  |  |  |  |  |  |  |  |
| SC/ST (Ref) |  |  |  |  |  |  |  |  |  |  |  |  |  |  |
| OBC |  | 1.15* |  | 1.2* |  | 1.26* |  | 1.05 |  | 1.28* |  | 1.16* |  | 1.05 |
| Others |  | 1.21* |  | 1.23* |  | 1.31* |  | 1.05 |  | 1.38* |  | 1.21* |  | 1.05 |
| **Residence** |  |  |  |  |  |  |  |  |  |  |  |  |  |  |
| Urban (Ref) |  |  |  |  |  |  |  |  |  |  |  |  |  |  |
| Rural |  | 1.02 |  | 1.09 |  | 1.13* |  | 1.07 |  | 1.1* |  | 0.95 |  | 1.04 |
| **Education level of Cluster** |  |  |  |  |  |  |  |  |  |  |  |  |  |  |
| Low (Ref) |  |  |  |  |  |  |  |  |  |  |  |  |  |  |
| Medium |  | 1.17* |  | 1.28* |  | 1.29* |  | 1.19* |  | 1.36* |  | 1.08* |  | 1.03 |
| High |  | 1.2* |  | 1.49* |  | 1.4* |  | 1.26* |  | 1.58* |  | 1.02 |  | 0.9 |
| **Wealth status of Cluster** |  |  |  |  |  |  |  |  |  |  |  |  |  |  |
| Low (Ref) |  |  |  |  |  |  |  |  |  |  |  |  |  |  |
| Medium |  | 1.17* |  | 1.16* |  | 1.09* |  | 1.15* |  | 1.25* |  | 1.15* |  | 1.19* |
| High |  | 1.28* |  | 1.27* |  | 1.16* |  | 1.13 |  | 1.36* |  | 1.25* |  | 1.53* |
| **Education level of District** |  |  |  |  |  |  |  |  |  |  |  |  |  |  |
| Low (Ref) |  |  |  |  |  |  |  |  |  |  |  |  |  |  |
| Medium |  | 1.08* |  | 1.1 |  | 1.16* |  | 1.05 |  | 1.14* |  | 1.09 |  | 0.97 |
| High |  | 1.03 |  | 1.07 |  | 1.2* |  | 0.92 |  | 1.21* |  | 1.03 |  | 0.78 |
| **Wealth status of District** |  |  |  |  |  |  |  |  |  |  |  |  |  |  |
| Low (Ref) |  |  |  |  |  |  |  |  |  |  |  |  |  |  |
| Medium |  | 1.01 |  | 0.92 |  | 0.92 |  | 1.07 |  | 0.97 |  | 1.06 |  | 1.15 |
| High |  | 1.04 |  | 0.97 |  | 0.87* |  | 1.05 |  | 1 |  | 1.13 |  | 1.29 |
| **Education level of State** |  |  |  |  |  |  |  |  |  |  |  |  |  |  |
| Low (Ref) |  |  |  |  |  |  |  |  |  |  |  |  |  |  |
| Medium |  | 1.04 |  | 1.39 |  | 1.21 |  | 1 |  | 1.11 |  | 0.72 |  | 1.6 |
| High |  | 1.13 |  | 2.22 |  | 1.47 |  | 1.79 |  | 1.1 |  | 0.47* |  | 0.53 |
| **Wealth status of State** |  |  |  |  |  |  |  |  |  |  |  |  |  |  |
| Low (Ref) |  |  |  |  |  |  |  |  |  |  |  |  |  |  |
| Medium |  | 1.01 |  | 0.76 |  | 0.88 |  | 0.93 |  | 0.9 |  | 1.36 |  | 0.83 |
| High |  | 1.06 |  | 0.5 |  | 0.74 |  | 0.58 |  | 1.24 |  | 2.49* |  | 2.22 |
| **Random Part** |  |  |  |  |  |  |  |  |  |  |  |  |  |  |
| State level variance | 0.048 | 0.054 | 0.170 | 0.158 | 0.065 | 0.127 | 0.097 | 0.088 | 0.071 | 0.232 | 0.106 | 0.092 | 0.337 | 0.242 |
| State VPC % | 1.3 | 1.5 | 4.5 | 4.3 | 1.7 | 3.7 | 1.9 | 1.9 | 1.9 | 6.5 | 2.5 | 2.2 | 5.0 | 3.7 |
| District level variance | 0.060 | 0.058 | 0.064 | 0.052 | 0.027 | 0.022 | 0.099 | 0.076 | 0.058 | 0.071 | 0.126 | 0.123 | 0.322 | 0.302 |
| District VPC % | 1.6 | 1.6 | 1.7 | 1.4 | 0.7 | 0.6 | 1.9 | 1.7 | 1.5 | 2.0 | 2.9 | 2.9 | 4.8 | 4.6 |
| Cluster level variance | 0.277 | 0.238 | 0.294 | 0.214 | 0.355 | 0.000 | 1.611 | 1.108 | 0.368 | 0.000 | 0.792 | 0.736 | 2.728 | 2.672 |
| Cluster VPC % | 7.5 | 6.5 | 7.7 | 5.8 | 9.5 | 0.0 | 31.6 | 24.3 | 9.7 | 0.0 | 18.4 | 17.4 | 40.9 | 41.1 |

**Note: * p<0.05**

**S10 Fig a-f:** Correlation between the district-level rates and within-district or between-cluster standard deviation of disability by its type in India, NFHS 2019-21. (a.) Hearing (b.) Speech (c.) Visual (d.) Mental (e.) Locomotor (f.) Other.

1. **Hearing (b) Speech**


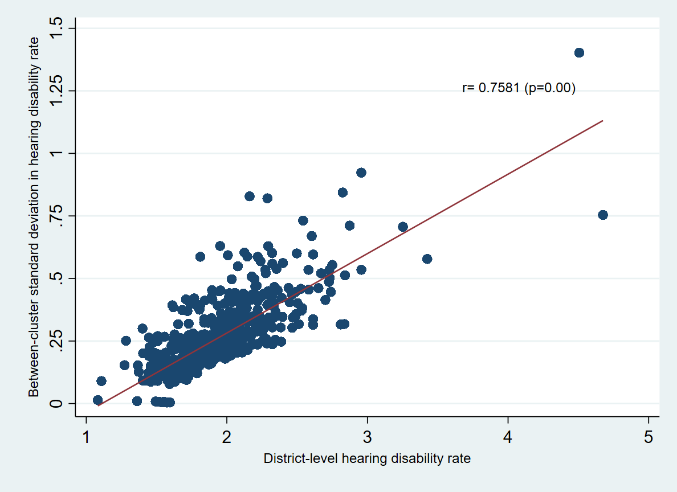

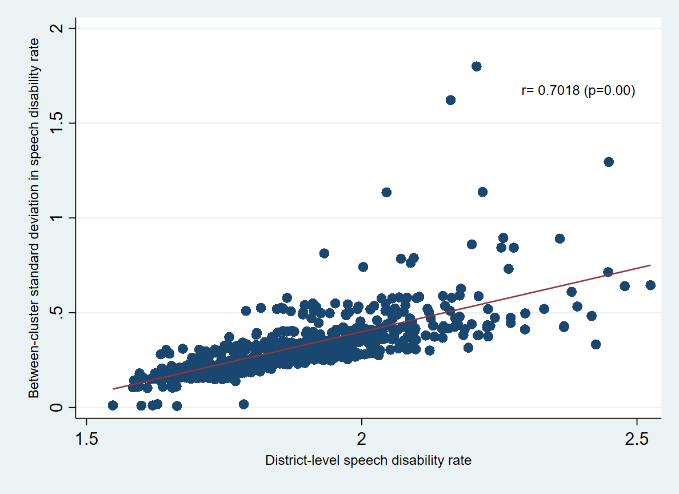


1. **Visual (d) Mental**


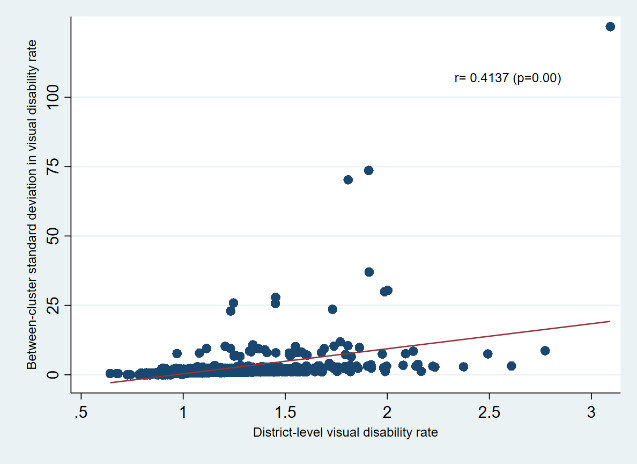

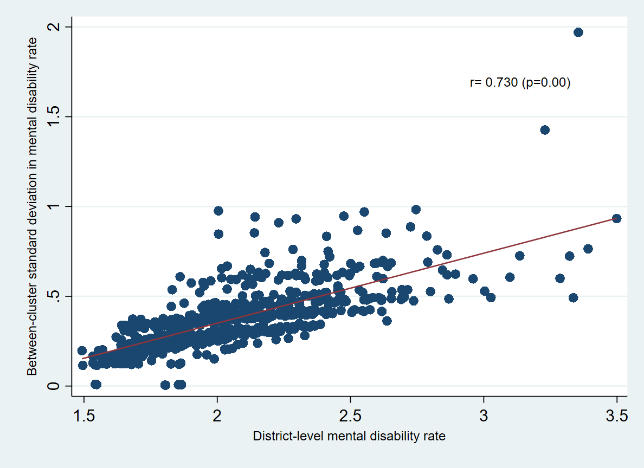


1. **Locomotor (f) Other**


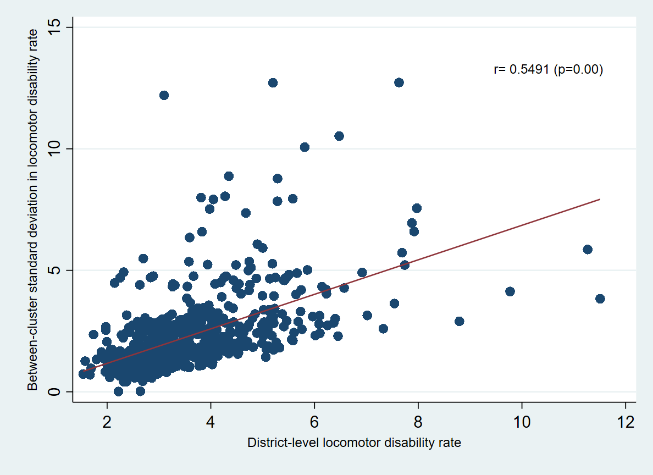

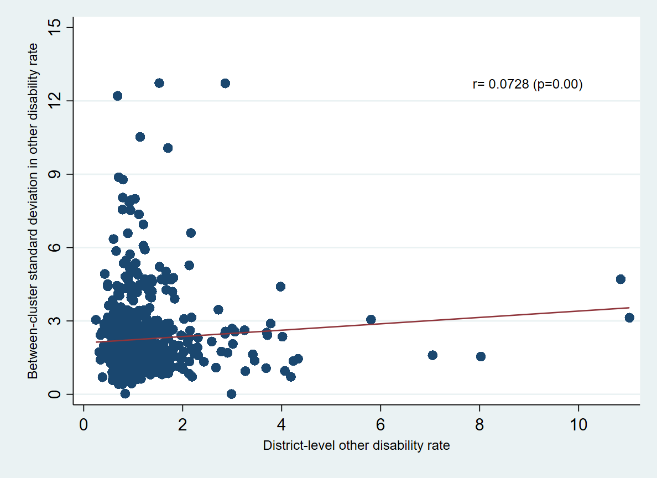

Supplement: Supplementary file 1 — Additional file 1: Figure S1. Schematic representation of the four-level hierarchical structure of the final analytic sample, NFHS 2019–21, India. Table S2. Distribution of number of districts, clusters, individuals, and disabled individuals, within 36 Indian states/union territories, NFHS 2019–21, India. Table S3. Description of various types of disability considered in NFHS 2019–21. Table S4. Sample distribution of participant’s characteristics in NFHS 2019–21, India. Table S5. Age-sex adjusted wealth-related inequalities in the prevalence of disability by its type in India, NFHS 2019–21. Table S6. Age-sex adjusted education-related inequalities in the prevalence of disability by its type in 18 + individuals of India, NFHS 2019–21. Figure S7. a-f: Concentration curves for different types of disability by education of 18 + individuals in India, NFHS 2019–21. (a.) Disability in any form (b.) Hearing (c.) Speech (d.) Visual (e.) Mental (f.) Locomotor. Figure S8. Multilevel logistic regression model showing association of covariates on disability by its type in India, NFHS 2019–21. Table S9. Four-level random intercept logit model showing risk factors associated with disability across its type in India, NFHS 2019–21. Figure S10. a-f: Correlation between the district-level rates and within-district or between-cluster standard deviation of disability by its type in India, NFHS 2019–21. (a.) Hearing (b.) Speech (c.) Visual (d.) Mental (e.) Locomotor (f.) Other. [file 12942_2024_363_MOESM1_ESM.docx]
